# Medical Graduates of the Present Season

**Published:** 1844-04-20

**Authors:** 


### RECORD OF MEDICAL SCIENCE.

### MEDICAL GRADUATES OF THE PRESENT SEASON.

| | |
|---|---|
| University of Pennsylvania, - - - - - | 153 |
| Jefferson Medical College, - - - - - | 117 |
| University of the City of N. Y., - - - - | 93 |
| Medical College of South Carolina, - - | 82 |
| Transylvania University, Lexington, Ky., - - | 59 |
| Louisville Institute, - - - - - - | 47 |
| University of Maryland, - - - - - - | 38 |
| Ohio Medical College, - - - - - - | 35 |
| College of Physicians and Surgeons, N. Y., - | 32 |
| Kemper College, - - - - - - - | 27 |
| Medical College, Richmond, Va., - - - - | 24 |
| Yale College, - - - - - - - - | 18 |
| Harvard University, Boston, - - - - - | 17 |
| Pennsylvania College, - - - - | 7 |

